# Data of RNA-seq transcriptomes of liver, bone, heart, kidney and blood in klotho mice at a pre-symptomatic state and the effect of a traditional Japanese multi-herbal medicine, juzentaihoto

**DOI:** 10.1016/j.dib.2022.108197

**Published:** 2022-04-22

**Authors:** Norihiro Okada, Kenshiro Oshima, Yuki Iwasaki, Akiko Maruko, Kenya Matsumura, Erica Iioka, Trieu-Duc Vu, Naoki Fujitsuka, Akinori Nishi, Aiko Sugiyama, Mitsue Nishiyama, Atsushi Kaneko, Kazushige Mizoguchi, Masahiro Yamamoto, Susumu Nishimura

**Affiliations:** a School of Pharmacy, Kitasato University, 1-15-1 Kitasato, Minami, Sagamihara, Kanagawa 252- 0373 Japan; b Foundation for Advancement of International Science (FAIS) , 3-24-16 Kasuga, Tsukuba, Ibaraki 305-0821 Japan; c Nagahama Institute of Bio-Science and Technology, Nagahama, Japan; d Tsumura Kampo Research Laboratories, Tsumura & CO., 3586 Yoshiwara, Ami-machi, Ibaraki 300- 1192 Japan; e Laboratory Animal Resource Center, University of Tsukuba, 1-1-1 Tennodai, Tsukuba, Ibaraki 305- 8575 Japan

**Keywords:** Intron retention, Pre-symptomatic state, Aging, RNA-seq, Alternative splicing, Japanese herbal medicine, Kampo

## Abstract

We performed RNA-seq analyses of mRNA isolated from five organs, liver, bone, heart, kidney and blood at the pre-symptomatic state of klotho mice with/without administration of a Japanese traditional herbal medicine, juzentaihoto (JTT). Data of differentially expressed genes (DEG) with/without JTT was included. Intron retention (IR) is an important regulatory mechanism that affects gene expression and protein functions. We collected data in which retained-introns were accumulated in a particular set of genes of these organs, and showed that among these retained introns in the liver and bone a subset was recovered to the normal state by the medicine. All of the data present changes of molecular events on the levels of metabolites, proteins and gene expressions observed at the pre- symptomatic state of aging in klotho mice with/without JTT. The research article related to this Data in Brief is published in GENE entitled as “Intron retention as a new pre-symptomatic marker of aging and its recovery to the normal state by a traditional Japanese herbal medicine”.

## Specifications Table

**Table:** 

Subject	Biological sciences, aging, molecular biology
Specific subject area	Alternative splicing, intron retention
Type of data	Figures and Table
How data were acquired	DNA sequencing Instrument: NovaSeq 6000 (Illumina) Program: NovaSeq Control Software v1.3.0 (Illumina) Library construction reagents: TruSeq Stranded mRNA Sample Prep Kit (illumina) Sequencing reagents: NovaSeq 6000 S4 Reagent Kit (illumina) Real-time PCR Instrument: StepOnePlus (Thermofisher) Reagent: Power SYBR® Green RNA-to-C_T_ 1-Step Kit (Thermofisher) Western Blot Homogenization: disposable homogenizer (Nippi BioMasher) Protein assay reagents: BCA protein assay (Thermo Fisher Scientific), ECL Prime Western Blotting Detection Reagents (GE Healthcare) Electrophoresis: myPower II 300 (ATTO) Detection program: ImageJ (NIH), ChemiDoc system (Bio-Rad)
Data format	Filtered raw reads (FASTQ)
Parameters for data collection	Male α-klotho knockout mice and wild-type mice were bred from 3.5 weeks after birth in a JTT-administered group and a non-administered group. At 7 weeks, mouse tissues were removed from each group and RNA was extracted. Sequencing library construction from mRNA was prepared according to the supplier's manual. Sequencing was performed the supplier's manual for paired-end sequencing.
Description of data collection	Mouse tissues were removed from each group and RNA was extracted using Pure Link RNA Mini kit (Invitrogen). Sequencing library construction for Illumina NovaSeq 6000 from mRNA was prepared according to the supplier's manual using TruSeq Stranded mRNA Sample Prep Kit (Illumina). Sequencing was performed the supplier's manual for paired-end sequencing (150 bases × 2) using NovaSeq 6000 and NovaSeq 6000 S4 Reagent Kit. Details were described in “Chapter 2 Experimental Design, Materials and Methods”.
Data source location	This data was collected and analyzed at school of pharmacy, Kitasato University, Kanagawa, Japan as well as at FAIS, Ibaraki, Japan. Mice were bred and dissected at CLEA Japan.
Data accessibility	Repository center: DNA Data Bank of Japan (DDBJ), DDBJ Sequence Read Archive (DRA) Data identification number: The accession numbers shown in Table 1. Direct URL to data: https://www.ncbi.nlm.nih.gov/bioproject/PRJDB7898
Related research article	N. Okada, K. Oshima, Y. Iwasaki, A. Maruko, K. Matsumura, E. Iioka, T.-D. Vu, N. Fujitsuka, A. Nishi, A. Sugiyama, M. Nishiyama, A. Kaneko, K. Mizoguchi, M. Yamamoto, S. Nishimura Intron retention as a new pre-symptomatic marker of aging and its recovery to the normal state by a traditional Japanese multi-herbal medicine, Gene (2021) 794:145,752. 10.1016/j.gene.2021.145752.

## Value of the Data

• The data provides that retained-introns accumulated in the pre-symptomatic state, and a subset of them was changed into the administration of traditional Japanese herbal medicine.
• The data provides proof-of-concept evidence related to the ancient Chinese statement proposing the medicine's usefulness for treating the pre-symptomatic state. Accordingly, it encourages all the researchers who are trying to get molecular evidence in the field of herbal medicines.
• The data propose that stress-dependent intron retention is a biomarker for pre-symptomatic and aging. This proposal is presented only using a limited set of organs, mainly klotho mice. To strengthen the generality, it is necessary to use many different organisms and their organs, including humans. This data will provide a basis for future research in the search for biomarkers of stress response and aging.

## 1 Data Description

**Fig. 1:**
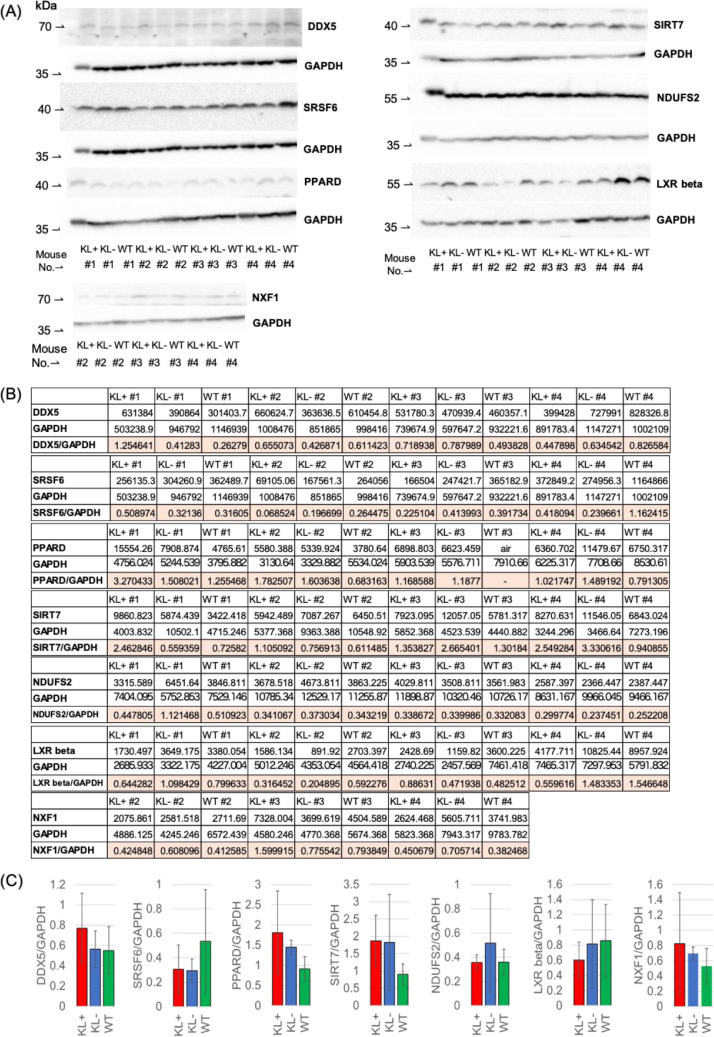
**Characterization of seven proteins.** (A) Images of seven representative membranes (DDX5, SRSF6, PPARD, SIRT7, NDUFS2, LXR and NXF1) by using Western blotting were shown. GAPDH used as a control. (B) Tables showed intensity values of each band. They were normalized by using those of GAPDH. (C) Bar plots showed quantitation of protein revels. From the left, the data for DDX5, SRSF6, PPARD, SIRT7, NDUFS2, LXR and NXF1 were shown. One-way ANOVA was used to calculate statistical significance. Expression levels did not change significantly in KL+, KL–, and WT. Error bars indicate mean ± standard deviations (*n* = 3 or 4).

**Fig. 2:**
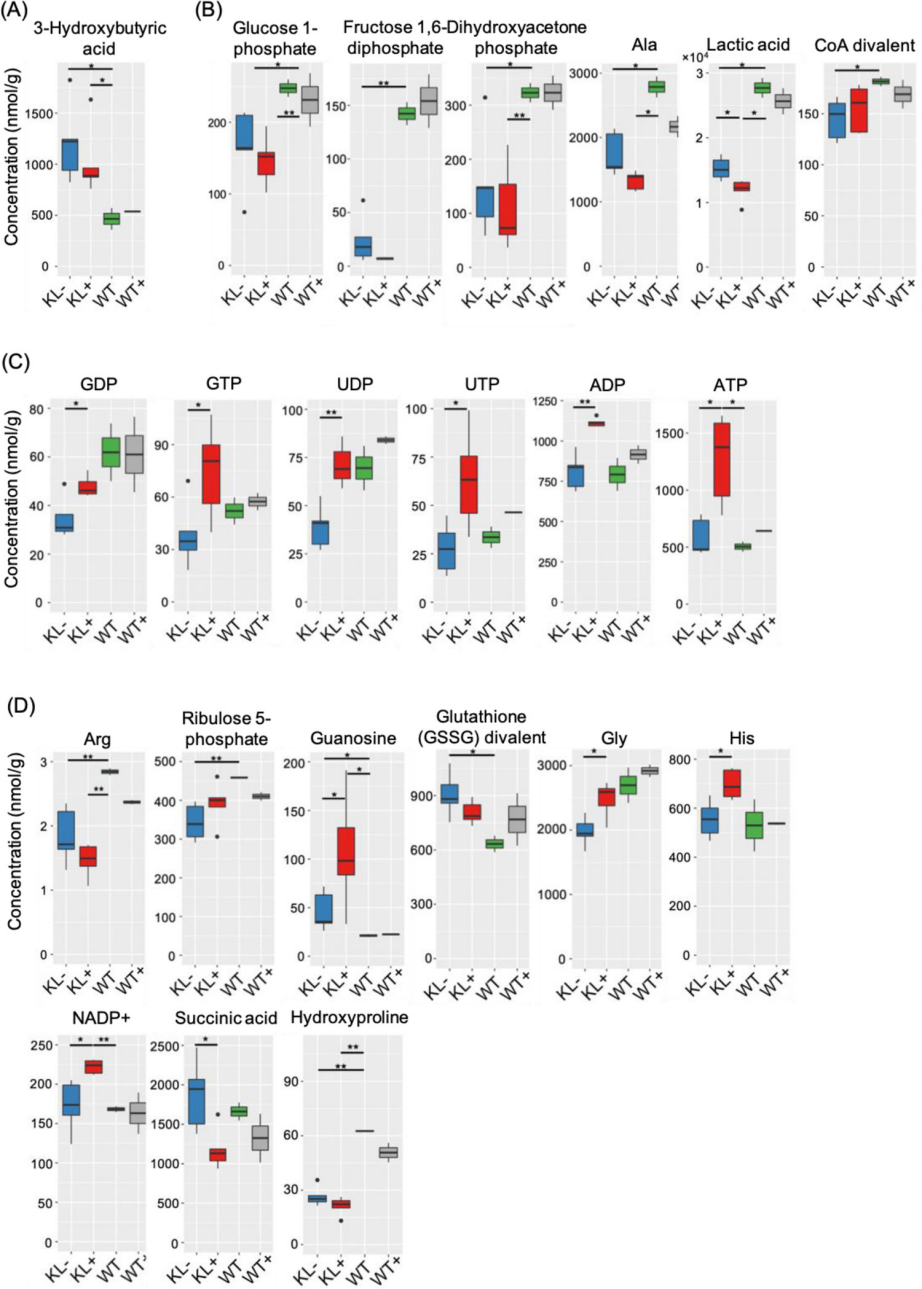
**A metabolome analysis showed that the klotho mice are undergoing a starvation-like condition at 7 weeks of age.** Based on a metabolome analysis of (A) 3-Hydoroxybutyric acid (3-HBA) and (B) other glycolysis-related metabolites, KL– data indicate that these mice are undergoing a starvation-like condition. (C) Based on the metabolome analysis about metabolites related to RNA precursors, the concentrations of several nucleotides were recovered in KL+ in comparison with KL– and WT. Data in (D) are those of 9 additional metabolites. *P < 0.05, **P < 0.01, unpaired Student's *t*-test.

**Fig. 3:**
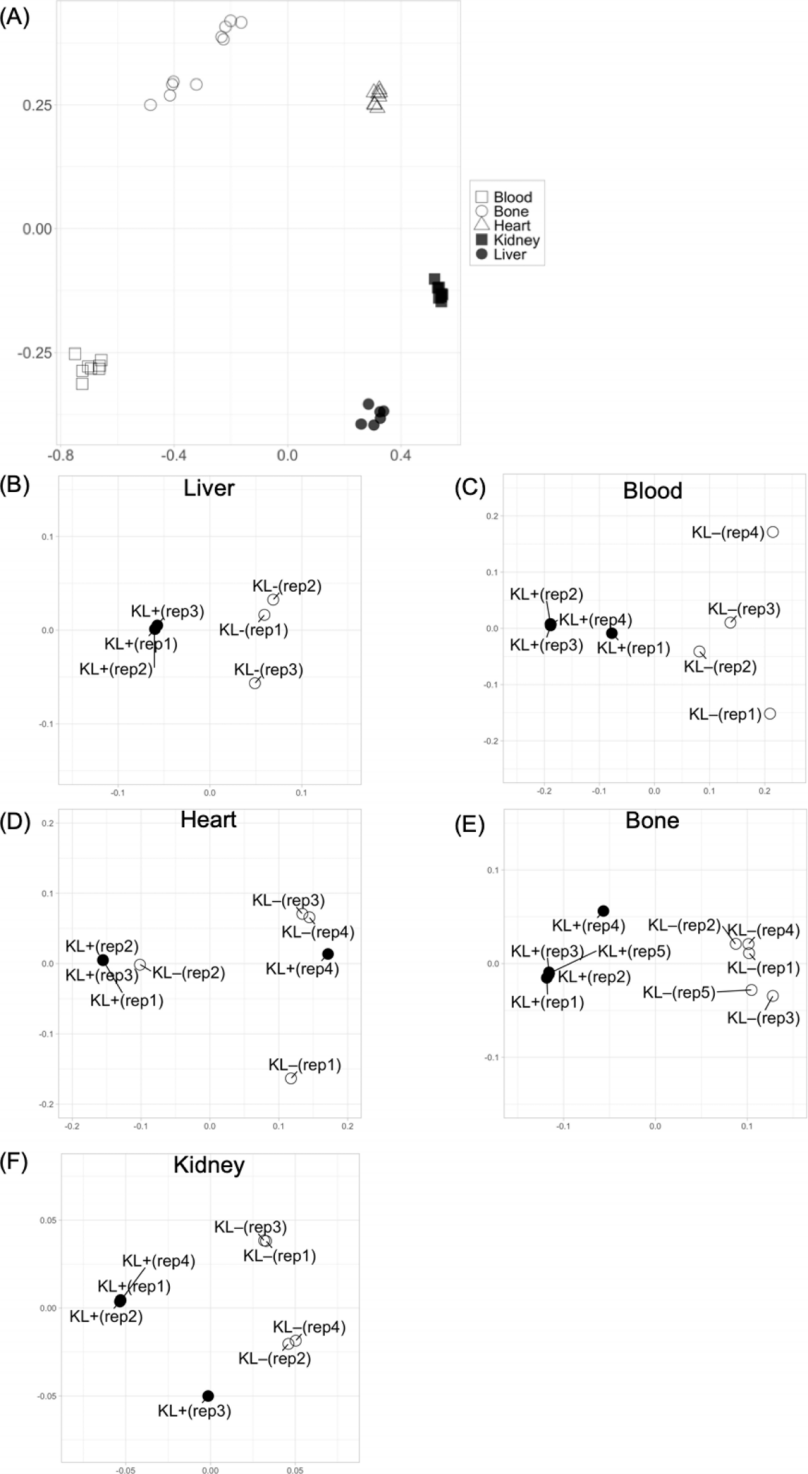
**Multi-dimensional scaling of transcriptomes of 5 organs in klotho mice.** (A) Each MDS shows well separation of transcriptomes to one another, reflecting integrity of transcriptomes from each organ (blood, bone, heart, kidney and liver). (B–F) Patterns of MDS using transcriptomes from liver (B), blood (C), heart (D), bone (E) and kidney (F) in klotho mice with JTT (KL+, filled circles) or without JTT (KL–, open circles).

**Fig. 4:**
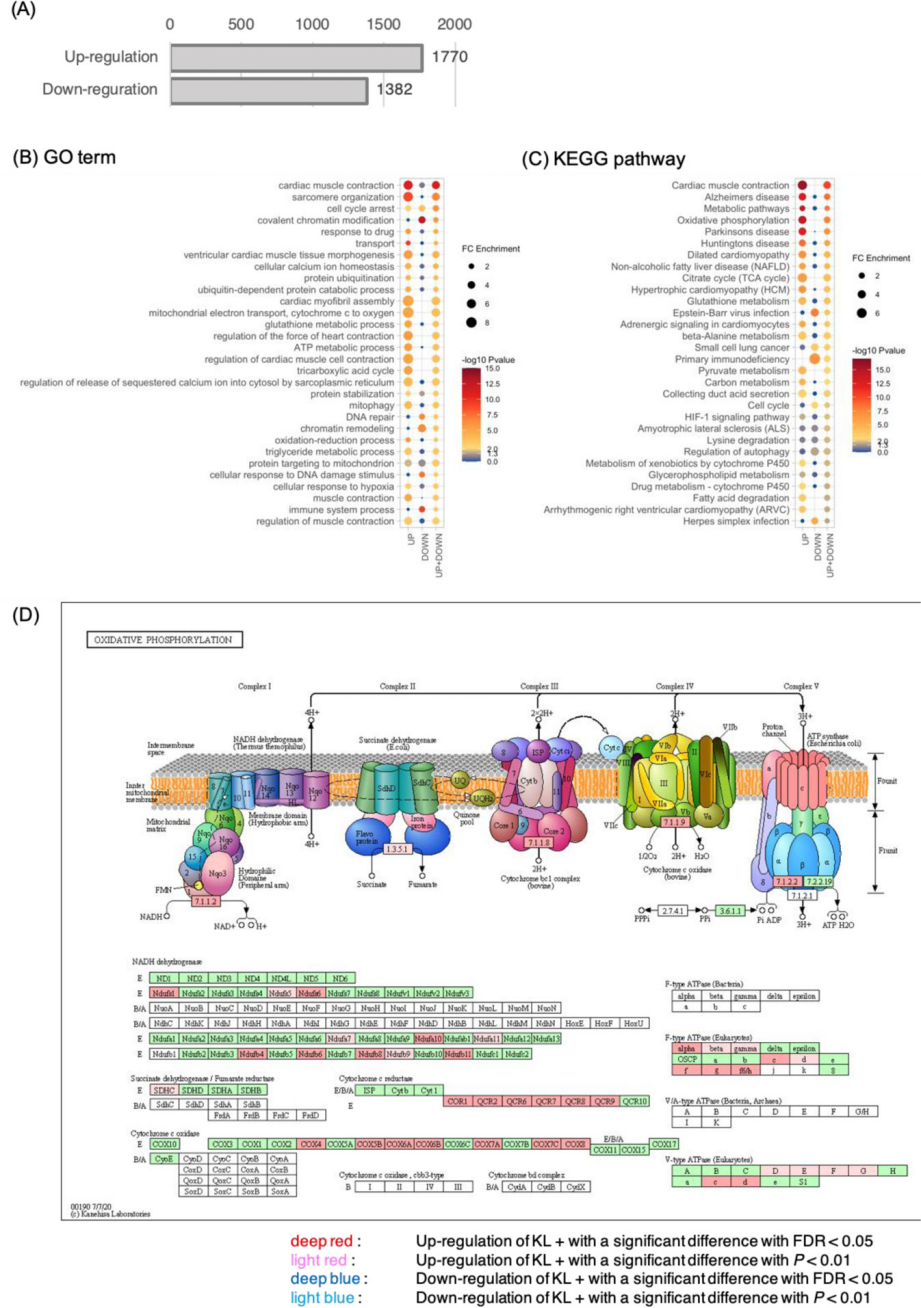
**JTT upregulates gene expressions related to mitochondria including oxidative phosphorylation in blood.** (A) The number of genes with a significant difference with *P* < 0.01 in the expression level between KL+ and KL– in blood. Up-regulation: The expression level is significantly increased in KL+. Down-regulation: The expression level is significantly decreased in KL+. Enrichment analyses of GO term (B) and KEGG pathway (C) for genes in that the expression levels were significantly up-regulated and/or down-regulated. The gradation of circle colours indicates the P value in the Fisher Exact test for the degree of enrichment. The circle size indicates the degree of enrichment by fold change. "UP" indicates the case of using the up-regulated 1770 genes in KL+, “DOWN” indicates the case of using the down-regulated 1382 genes in KL+. "UP+DOWN" indicates the case in which a total of 3152 genes were used. (D) Genes in KEGG pathway of oxidative phosphorylation showing upregulation and downregulation by administration of JTT in KL+. Coloration in relation to statistical values was shown in the legend in (D).

**Fig. 5:**
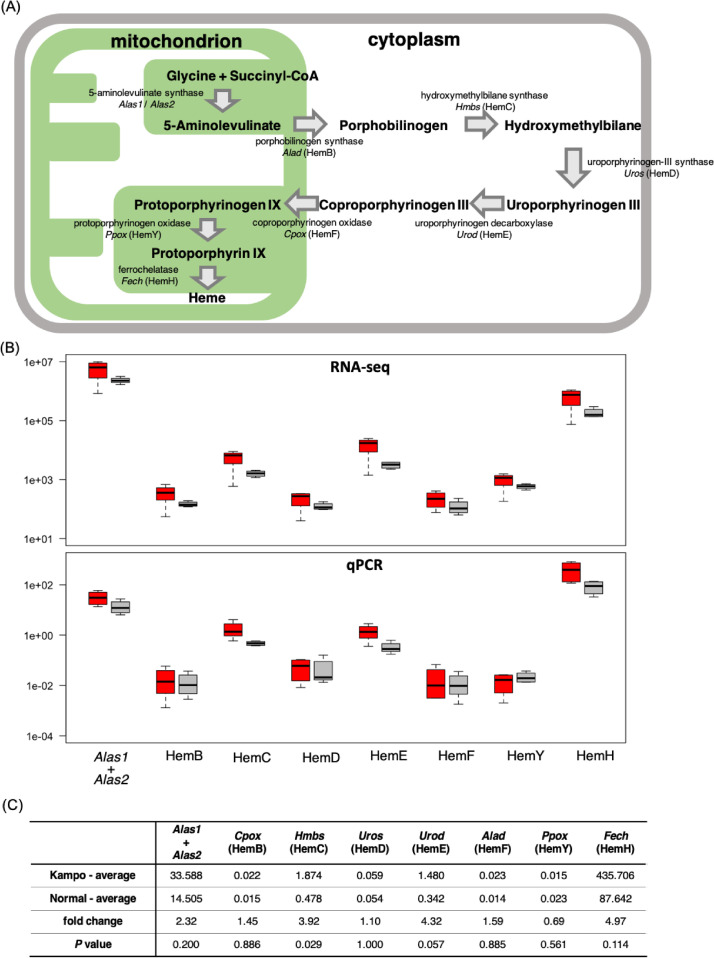
**The levels of gene expression related to the heme biosynthetic pathway were compared between KL+ and KL–, showing that JTT upregulated genes involved in heme synthesis in blood.** (A) The heme biosynthetic pathway is initiated by the synthesis of the amino acids glycine and succinyl CoA. Heme is synthesized via 5-aminolevulinate, porphobilinogen, hydroxymethylbilane, uroporphyrinogen III, coproporphyrinogen III, protoporphyrinogen IX, and protoporphyrin IX as intermediates. (B) Comparison of RNA-seq data (upper) and q-PCR data (lower) of nine gene expression levels involved with the heme biosynthesis pathway. We compared the expression of mRNA from KL+ (red) and KL– (gray). (C) The data by q-PCR for the expression of mRNA for nine genes involved in hem biosynthesis and the fold change for the average value of the sample with JTT (Kampo-average) to the average value of the sample without JTT (Normal-average).

**Fig. 6:**
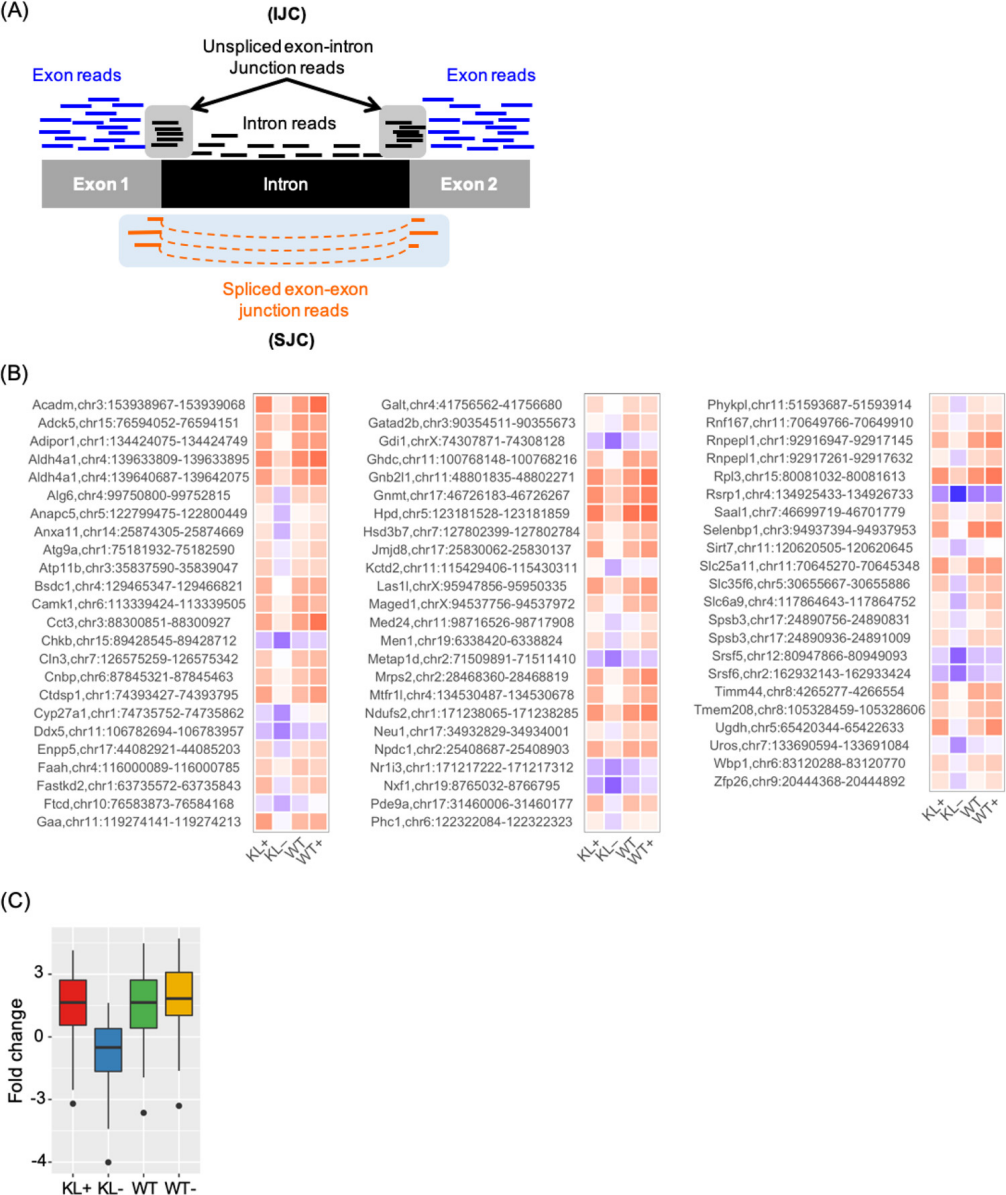
**The quantification of IR events from mRNA-seq data using rMATS showed the presence of the 70 complete recovery loci by administration of JTT in the liver.** (A) The IJCs represent the reads containing the intron sequence at the junction. The SJCs represent the reads without intron sequences at the junction. The extent of IR in WT with JTT is almost the same as that of WT in the seventy loci of complete recovery in the liver. The data in (B) is the same as Fig. 5B & C in the related research paper except for the addition of WT+. Heat map illustration of FC values of loci in KL+, KL–, WT, and WT+ for the 70 “complete recovery” loci. (C) Boxplots of the FC values of the 70 “complete recovery” loci.

**Fig. 7:**
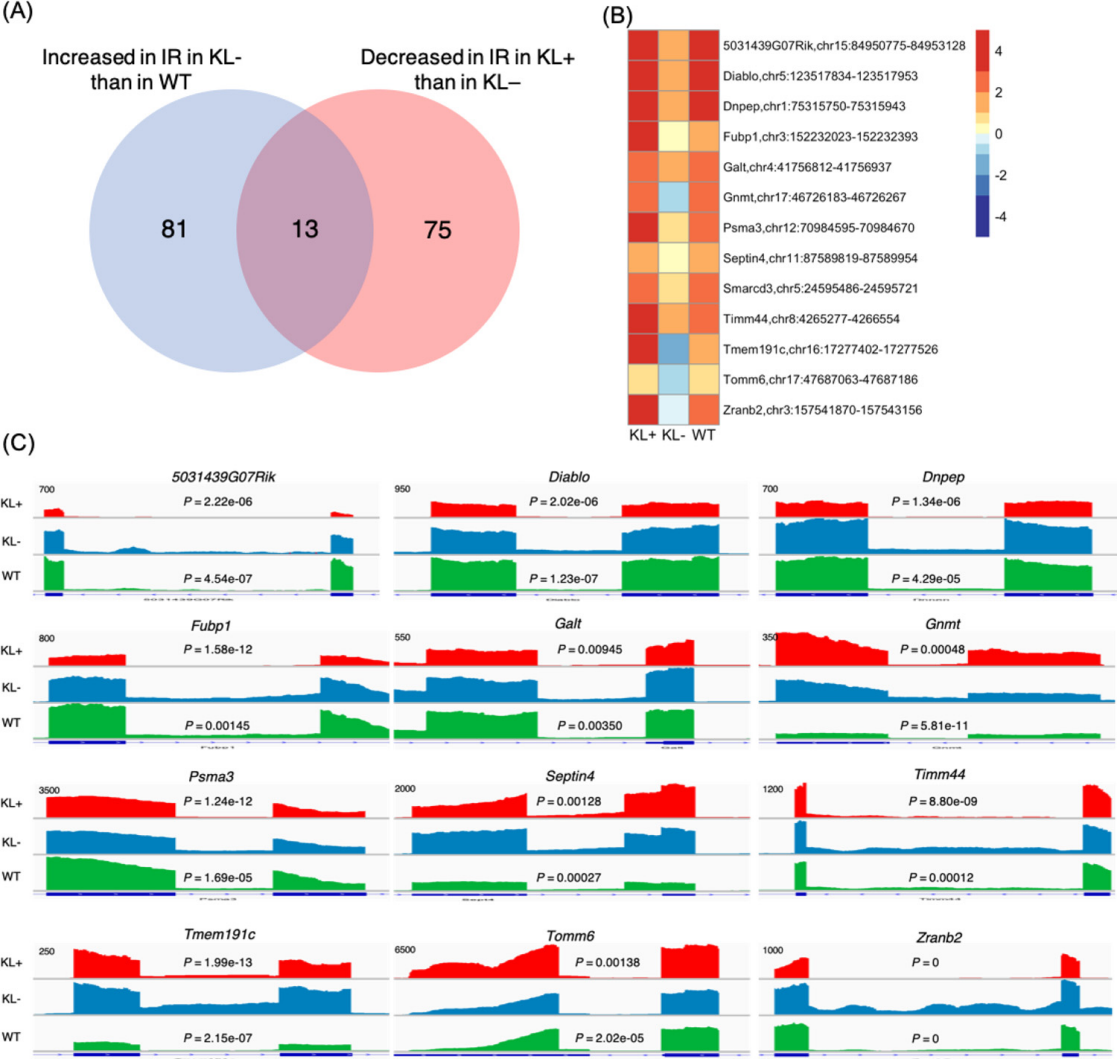
**The 13 complete recovery loci by administration of JTT in the bone**. (A) A Venn diagram which describes overlapping between 93 genes whose intron was increased in KL– compared with WT (i.e. aging) and 88 genes whose intron was recovered to the healthy state by JTT in KL. Thus the 13 overlapping genes were isolated. (B) Heatmap of FC values of loci in KL+, KL– and WT for the 13 “complete recovery” loci. (C) The mapping results of intron loci for *5031439G07Rik, Diablo, Snpep, Fubp1, Galt, Gnmt, Psma2, Spetin4, Timm44, Tmem191c, Tomm6* and *Zranb2* from KL+, KL–, and WT are shown using IGV.

**Fig. 8:**
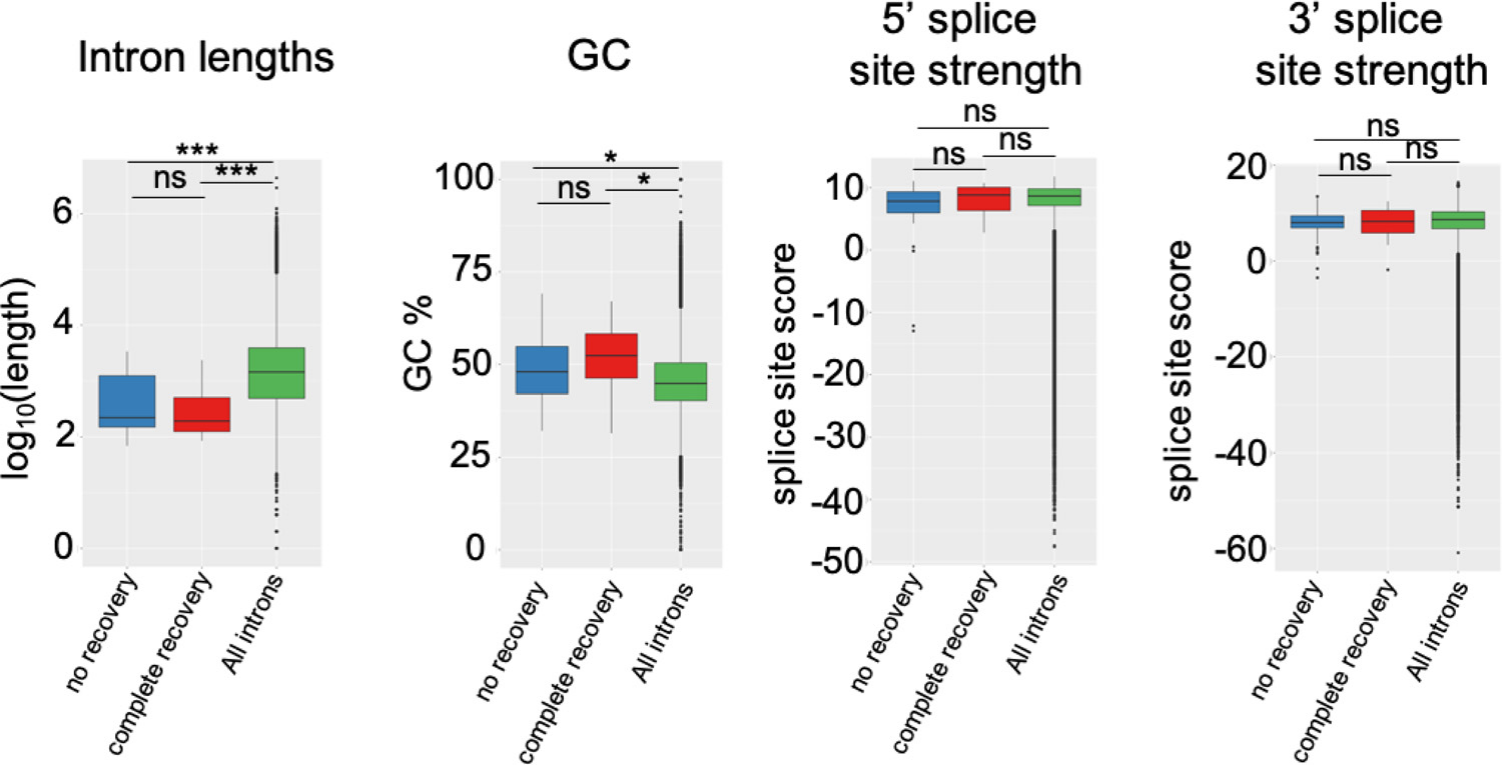
**IR loci have their own characteristics.** Boxplots showing intron lengths, GC% of intron sequences, and the strength score of 5´/3’ splice sites compared among three groups of introns, namely “no recovery”, “complete recovery” and “All introns” (254,005 loci) in the bone. Statistical analyses are unpaired Student's t tests, and significance is annotated as ∗: *P* < 0.05,∗∗: *P* < 0.01, ∗∗∗: *P* < 0.001.

**Fig. 9:**
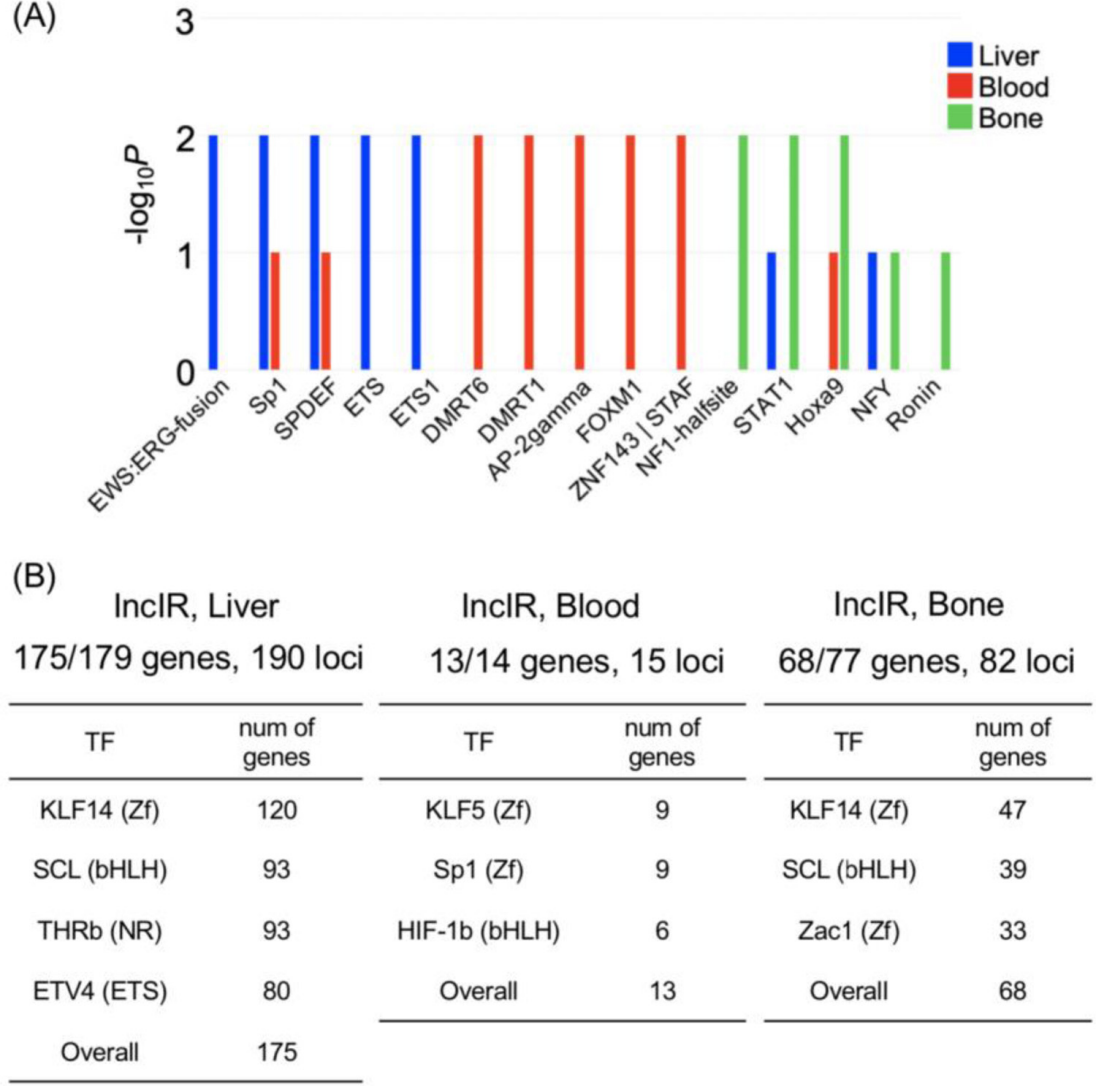
**A very few transcription factors (TFs) may control genes with IncIR**. (A) Bar graph showing TF-binding motifs that are significantly enriched in genes with IncIR from liver, blood and bone. (B) The table shows the combinations of TF-binding motifs that can cover more genes in the liver, blood and bone, respectively.

In relation to the related research article, we collected the data which represented the pre-symptomatic state (7 weeks of age) of klotho mice and the recovery from this state to the healthy state on the molecular level by administration of traditional Japanese herbal medicine, juzentaihoto (JTT) [1], [2], [3]. Western blot analysis showed that the pattern of protein expressions at this state is not so much changed from that of wild type (Fig. 1). Metabolome analysis showed that the state is under a starvation-like condition (Fig. 2). RNA was isolated from five organs (liver, blood, kidney, heart and bone) and their transcriptomes were determined by using RNA-seq. Integrity of RNA-seq data was confirmed by MDS analysis (Fig. 3). Analyses of differentially expressed genes in the blood between klotho mice with JTT (KL+) and those without JTT (KL–) showed that JTT upregulated genes related to mitochondria including oxidative phosphorylation (Fig. 4). Similarly, genes involved in heme biosynthesis were upregulated by administration of JTT in the blood of KL+ (Fig. 5). By using rMATS software (Fig. 6A), we showed that retained introns were accumulated even at the pre-symptomatic state, a subset of which were recovered to the healthy state by JTT in the liver. In the case of wild type, administration of JTT does not change the gene expression profile in WT+ with that of WT– (Fig. 6B & 6C). Fig. 7 shows that JTT can completely recover the 13 intron-retained loci in the bone to the healthy state by administration of JTT. Fig. 8 shows their own characteristics of IR loci in the case of genes from the bone, in terms of intron length, GC% of intron sequences and the strength score of 5´/3´ splice sites. Fig. 9 shows that a very few TFs may control genes with increased intron (IncIR) in liver, blood and bone.

**Table 1:** List of accession numbers for next generation sequencing data in DNA Data Bank of Japan (DDBJ), DDBJ Sequence Read Archive (DRA).

	KL+ (KL with JTT)	KL– (KL without JTT)	WT (WT without JTT)
Liver	DRR167982 (KL liver with JTT rep1) DRR167983 (KL liver with JTT rep2) DRR167984 (KL liver with JTT rep3)	DRR167985 (KL liver without JTT rep1) DRR167986 (KL liver without JTT rep2) DRR167987 (KL liver without JTT rep3)	DRR167988 (WT liver without JTT rep1) DRR167989 (WT liver without JTT rep2) DRR167990 (WT liver without JTT rep3)

Bone	DRR259222 (Kl bone with JTT rep1) DRR259223 (Kl bone with JTT rep2) DRR259224 (Kl bone with JTT rep3) DRR259225 (Kl bone with JTT rep4) DRR259226 (Kl bone with JTT rep5)	DRR259227 (Kl bone without JTT rep1) DRR259228 (Kl bone without JTT rep2) DRR259229 (Kl bone without JTT rep3) DRR259230 (Kl bone without JTT rep4) DRR259231 (Kl bone without JTT rep5)	DRR259232 (WT bone without JTT rep1) DRR259233 (WT bone without JTT rep2) DRR259234 (WT bone without JTT rep3) DRR259235 (WT bone without JTT rep4) DRR259236 (WT bone without JTT rep5)

Kidney	DRR259237 (WT kidney without JTT rep1) DRR259238 (WT kidney without JTT rep2) DRR259239 (WT kidney without JTT rep3) DRR259240 (WT kidney without JTT rep4)	DRR259241 (Kl kidney with JTT rep1) DRR259242 (Kl kidney with JTT rep2) DRR259243 (Kl kidney with JTT rep3) DRR259244 (Kl kidney with JTT rep4)	DRR259245 (Kl kidney without JTT rep1) DRR259246 (Kl kidney without JTT rep2) DRR259247 (Kl kidney without JTT rep3) DRR259248 (Kl kidney without JTT rep4)

Heart	DRR290048 (KL heart with JTT rep1) DRR290049 (KL heart with JTT rep2) DRR290050 (KL heart with JTT rep3) DRR290051 (KL heart with JTT rep4)	DRR290052 (KL heart without JTT rep1) DRR290053 (KL heart without JTT rep2) DRR290054 (KL heart without JTT rep3) DRR290055 (KL heart without JTT rep4)	DRR290056 (WT heart without JTT rep1) DRR290057 (WT heart without JTT rep2) DRR290058 (WT heart without JTT rep3) DRR290059 (WT heart without JTT rep4)

Blood	DRR290060 (KL blood with JTT rep1) DRR290061 (KL blood with JTT rep2) DRR290062 (KL blood with JTT rep3) DRR290063 (KL blood with JTT rep4)	DRR290064 (KL blood without JTT rep1) DRR290065 (KL blood without JTT rep2) DRR290066 (KL blood without JTT rep3) DRR290067 (KL blood without JTT rep4)	DRR290068 (WT blood without JTT rep1) DRR290069 (WT blood without JTT rep2) DRR290070 (WT blood without JTT rep3) DRR290071 (WT blood without JTT rep4)

## 2 Experimental Design, Materials and Methods

### 2.1 Experimental design

α-klotho knockout (*Kl*^−/−^ Jcl, klotho mice) mice have the phenotype of human progeria, with a lifespan of approximately 10 weeks, and are a model mouse for aging studies. Klotho mice (*N* = 10) and wild-type (WT, *N* = 10) 3-week-old male mice were purchased from CLEA Japan. Each mouse was acclimated for 3 days in a vinyl isolator at the breeding facility in CLEA Japan. Then, klotho mice and WT mice were bred separately in a group fed with juzentaihoto (JTT)-containing feed and a normal diet group, respectively. One group was fed CE-2 containing 0.5% (w/w) JTT powder and the other group was fed CE-2 without JTT. JTT is a traditional Japanese herbal medicine, the powdered extract of which is supplied by Tsumura (Tokyo, Japan), and is made by spray-drying boiling extracts from a mixture of 10 crude medicines. JTT contains 10.52% Astragali radix, 10.52% Cinnamomi cortex, 10.52% Rehmanniae radix, 10.52% Paeoniae radix, 10.52% Cnidii rhizome, 10.52% Atractylodis lanceae Rhizome, 10.52% Angelicae Ginseng, and 5.32% Glycyrrhizae radix. CE-2 is a general feed for rodents that contains vegetable protein as the main ingredient, as well as animal protein, fat, fiber, carbohydrates, vitamins, and minerals. Mice in each group were dissected at 7 weeks of age according to the animal ethics regulations, and liver (3 in each group), heart (4 in each group), bone (5 in each group), and kidney (5 in each group) were removed; those for RNA sequencing were soaked in RNALater and stored in the freezer, and a portion of liver tissue was frozen intact for metabolomic analysis. Whole blood (0.8 mL) was collected from the tail vein using a syringe containing 8–12 µL of EDTA-2 K (4 in each group). Then, equal volumes of PBS buffer and 3 vol of TRIzol (Thermo Fisher Scientific) were added, dispensed into tubes, and stored frozen. The experimental procedures using animals were approved by the Laboratory Animal Committee of CLEA Japan.

### 2.2 Western blot analysis (related to Fig. 1)

About 30 mg of mouse liver was homogenized using a disposable homogenizer (Nippi BioMasher) with RIPA buffer (150 mM NaCl, 50 mM Tris, 10 mM NaF, 1% NP-40, 0.5% deoxycholic acid sodium monohydrate, 0.1% SDS, pH 7.4) of 500 uL with protease inhibitor cocktail (FUJIFILM Wako, Osaka,Japan).

The homogenate solutions were centrifuged at 10,000 × *g* for 20 min at 4 °C. The protein concentration of the supernatant was measured with a Poerce BCA protein assay kit (Thermo Fisher Scientific). The supernatant was diluted to equalize the protein concentrations, and DTT (FUJIFILM Wako) was added to make the final concentration of 0.2 M. Then, 4 × SDS sample buffer (6% SDS, 40% glycerol, 0.4% bromophenol blue, 250 mM Tris, pH 6.8) was added and boiled at 95 °C for 5 min. The prepared protein samples (30 µg/well) were separated by electrophoresis on SDS-PAGE (10% gel) for 1–2 h at 20 mA/gel. After electrophoresis, they were transferred onto PVDF membranes (GE Healthcare) using wet transfer for 2 h at 100 mA on ice. The membranes were blocked with 5% skim milk in TBST (20 mM Tris, 150 mM NaCl, containing 0.05% Tween-20, pH 7.4), blotted with the antibodies described below. Primary antibodies used in this study were anti-DDX5, p68 (10,804–1-AP, Proteintech) 1:300, anti-NDUFS2 (GTX114924, GeneTex) 1:4000, anti-PPARD (10,156–2-AP, Proteintech) 1:500, anti-SIRT7 (12,994–1-AP, Proteintech) 1:1000, anti-NXF1 (10,328–1-AP, Proteintech) 1:500, anti-SRSF6 (11,772–1-AP, Proteintech) 1:2000, anti-LXR beta (ab28479, Abcam) 1:500, and anti-GAPDH (sc-32,233, Santa Cruz Biotechnology) 1:1000 in TBST with 5% milk.. After incubation with primary antibodies at 4 °C overnight, the membranes were washed three times for 5 min each in TBST. For detection, we used the secondary antibodies peroxidase-conjugated anti−rabbit IgG (SA00001–2, Proteintech) 1:16,000 or anti–mouse IgG (sc-516,102, Santa Cruz Biotechnology) 1:10,000 in TBST for 1 h at room temperature. After incubation, the membranes were washed three times in TBST for 10 min each, and protein bands were detected using enhanced chemiluminescence (ECL) Prime Western Blotting Detection Reagents (GE Healthcare). One of the membranes was probed with anti-GAPDH, and used as the loading control for other blots in each experiment. The signal intensity was quantified using ImageJ software (NIH, Bethesda, MD) or a ChemiDoc system (Bio-Rad). Western blots were repeated at least three times with different animals, and representative blots are shown.

### 2.3 Measurement of metabolites (related to Fig. 2)

Metabolome measurements and data processing were carried out through a facility service at Human Metabolome Technologies Inc., Tsuruoka, Japan. 50 mg of frozen liver tissue was crushed with 1500 µL of 50% acetonitrile/Milli-Q water containing an internal standard (Solution ID: 304–1002, Human Metabolome Technologies, Inc.) at 0 °C in a tissue homogenizer (Micro Smash MS100R, Tomy Digital Biology Co., Ltd., Tokyo, Japan). The homogenate was centrifuged at 2300 × *g* at 4 °C for 5 min. Then, 800 µL of the upper aqueous layer was filtered through a Millipore filter (5-kDa cutoff) at 9100 × *g* at 4 °C for 120 min to remove proteins. The filtrated solution was concentrated by centrifugation and resuspended in 50 µL of Milli-Q water.

Metabolome analysis was performed by capillary electrophoresis−time-of-flight mass spectrometry (CE-TOFMS) analysis. CE-TOFMAS experiments were performed using Agilent CE-TOFMAS system (Agilent Technologies Inc., Santa Clara, CA, USA). Separations were performed with the aid of a capillary filled with fused silica (50 mm internal diameter  ×  80 cm total length) filled with 1 M formic acid or 50 mM ammonium acetate (pH 8.5) as the electrolyte for cation or anion analyses, respectively. The applied voltage for separation was set at 30 kV. MS spectra were scanned from *m/z* 50 to 1000. MS was conducted in negative ionization mode with 3500 V for anion analysis and in positive ionization mode with 4000 V for cation analysis.

Data processing for peak picking, peak alignment, metabolite annotation, and peak integration was performed using MasterHands™(ver.2.17.1.11)(Institute for Advanced Biosciences, Keio University, JAPAN).

### 2.4 RNA extraction, RNA-seq, quality check, filtering of RNA-seq data and mapping analysis (related to Fig. 3)

Total RNA was extracted using the Pure Link RNA Mini kit (Invitrogen, MA, USA) according to the manufacture's protocol. Briefly, 600 μL of lysis buffer and 900 μL of TRIzol (Thermo Fisher Scientific) were added to 30 mg of individual tissue samples (liver, heart and kidney), and homogenized. After incubation at room temperature for 10 min, the samples were centrifuged at 12,000 × *g* for 15 min, the supernatant was vortexed with 200 μL of 1-Bromo-3-chloropropane (BCP) and centrifuged at 12,000 × g for 15 min. For blood samples, 200 μL of BCP was added per 1.5 mL of each blood samples (with PBS buffer and TRIzol), vortexed, and centrifuged at 12,000 × *g* for 15 min. The supernatant was treated with an equal volume of 70% ethanol and was purified by using column cartridge. During the washing procedure, the sample was treated with DNase (Invitrogen) on the column. After washing, RNA was eluted with 50–100 μL of RNase-Free Water. To confirm RNA quality, RNA concentration was measured using qubit (Thermo Fisher Scientific) and RIN values were confirmed using TapeStation (Agilent Technologies, CA, USA). For RNA-seq, RIN values of 6 or higher were used. RNA library was constructed by using a TruSeq Stranded mRNA Sample Prep kit (Illumina, CA, USA). Paired-end sequencing (150 *b* × 2) was outsourced to Takara Bio (Shiga, Japan) using a NovaSeq 6000 system (Illumina).

Quality filtering was used to examine the RNA sequences obtained from the next generation sequencers. First, the adapter sequences for Illumina that were ligate to prepare the sequencing library using cutadpt software v.1.16 were trimmed. (Optional parameters: -m 30 -b GATCGGAAGAGCACACGTCTGAACTCCAGTCAC -b AGATCGGAAGAGCGTCGTGTAGGGAAAGTGT) [4]. The poly(A) sequence was then trimmed using the fastx_clipper software supplied with the fastx toolkit software package v.0.0.14 [5]. were trimmed and short sequences that were less than 30 bases after trimming were removed using fastq_quality_trimmer software (Optional parameters: -t 20 -l 30 -Q 33) and fastq_quality_filter software (Optional parameters: -q 20 -p 80 -Q 33) included in the fastx toolkit software package. In the previous process, reads with one of the pairs missing were removed using Trimomatic v.0.38 software (Optinal parameters: MINLEN:30) [6]. Next, reads containing mouse rRNA and phiX standard sequences were detected and removed using Bowtie 2 v.2.3.4.1 software (parameters: default) [7]. The removal of unpaired reads was then performed again using bam2fastq software [8]. After these quality filtering, 20 million reads each of forward and reverse sequences per sample were mapped to the mouse genome sequence build GRCm38 using Tophat v2.1.1 (parameters: default) [9]. Mouse genome sequences were downloaded from the iGenomes of Illumina website [10]. Multiple mapping reads were then removed from the output BAM files using samtools software (Optional parameter: samtools view -q 4) [11]. Uniquely mapped reads by gene annotation (Ensembl release 81) were counted using FeatureCounts v.1.6.2 software [12]. The counted raw RNA expression values were normalized by the Trimmed mean of M values (TMM) method [13] using the EdgeR library [14] in R v.3.5.0, and used for expression analysis.

### 2.5 Analysis of alternative splicing by using rMATS (related to Fig. 6)

Intron loci with significantly different patterns (*P* < 0.01) of intron retention were detected using rMATS v.4.0.2 software [15] (Optional parameters: –cstat 0.05 -t paired –readLength 150 –variable-read-length). The combinations used in the calculations are KL+/KL–, KL+/WT, and KL–/WT, where “KL+” indicates JTT-treated Klotho mice, “KL–” indicates JTT-non-treated Klotho mice, and “WT” indicates wild-type mice. Statistical significance was tested using the number of skip junctions (SJCs) and inclusion junctions (IJCs) calculated by rMATS at the corresponding loci. Finally, we used the Integrative Genomics Viewer [16] to confirm our mapping results to intron loci.

### 2.6 Splice site score (related Fig. 8)

To evaluate splice site intensity at intron retention loci, MaxEntScan (parameters: default) [17] was used to calculate maximum entropy scores for 5 ´ and 3 ´ splice sites.

### 2.7 Motif analysis (related Fig. 9)

Protein binding motifs in mRNA were compared to RNA binding proteins and their associated motifs in the ATtRACT database [18]. Transcription factor binding sites were detected using HOMER software [19]. All detected motifs were compared by the Fisher's test.

## Ethics Statement

Experimental procedures using animals were carried out with approval from the Laboratory Animal Committee of CLEA Japan.

## CRediT Author Statement

**Norihiro Okada:** Conceptualization, Data curation, Formal analysis, Funding acquisition, Investigation, Methodology, Project administration, Supervision, Validation, Writing – original draft, Writing – review & editing; **Kenshiro Oshima:** Data curation, Formal analysis, Writing – review & editing; **Yuki Iwasaki:** Data curation, Formal analysis, Validation; **Akiko Maruko:** Data curation, Formal analysis, Validation; **Matsumura Kenya:** Data curation, Formal analysis, Validation; **Erica Iioka:** Data curation, Formal analysis, Validation; **Trieu-Duc Vu:** Data curation, Formal analysis, Validation; **Naoki Fujitsuka:** Writing – original draft, Data curation, Formal analysis, Validation; **Akinori Nishi:** Data curation, Formal analysis, Validation; **Aiko Sugiyama:** Data curation, Formal analysis, Validation; **Mitsue Nishiyama:** Data curation, Formal analysis, Validation; **Atsushi Kaneko:** Writing – original draft, Data curation, Formal analysis, Validation; **Kazushige Mizoguchi:** Conceptualization, Data curation, Formal analysis, Validation; **Masahiro Yamamoto:** Conceptualization, Writing – original draft, Data curation, Formal analysis, Validation; **Susumu Nishimura:** Conceptualization, Data curation, Formal analysis, Validation.

## Declaration of Competing Interest

The authors declare that they have no known competing financial interests or personal relationships that could have appeared to influence the work reported in this paper.

## Data Availability

No data was used for the research described in the article.

## References

[1] Saiki I. (2000). A Kampo medicine "Juzen-taiho-to"–prevention of malignant progression and metastasis of tumor cells and the mechanism of action. Biol. Pharm. Bull..

[2] Okada N., Oshima K., Iwasaki Y., Maruko A., Matsumura K., Iioka E., Vu T.-.D., Fujitsuka N., Nishi A., Sugiyama A., Nishiyama M., Kaneko A., Mizoguchi K., Yamamoto M., Nishimura S. (2021). Intron retention as a new pre-symptomatic marker of aging and its recovery to the normal state by a traditional Japanese multi-herbal medicine. Gene.

[3] UNESCO Huang Di Nei Jing (2011).

[4] Martin M. (2011). Cutadapt removes adapter sequences from high-throughput sequencing reads. EMBnet. J..

[5] FASTX-Toolkit website, http://hannonlab.cshl.edu/fastx_toolkit/index.html. Accessed June 1, 2021.

[6] Bolger A.M., Lohse M., Usadel B. (2014). Trimmomatic: a flexible trimmer for Illumina sequence data. Bioinformatics.

[7] Langmead B., Salzberg S.L. (2012). Fast gapped-read alignment with Bowtie 2. Nat. Methods..

[8] bam2fastq software, HudsonAlpha Discovery website: https://gslweb.discoveryls.com/information/software/bam2fastq. Accessed June 1, 2021.

[9] Kim D., Pertea G., Trapnell C., Pimentel H., Kelley R., Salzberg S.L. (2013). TopHat2: accurate alignment of transcriptomes in the presence of insertions, deletions and gene fusions. Genome Biol.

[10] iGenomes website - ready-to-use reference sequences and annotations, https://support.illumina.com/sequencing/sequencing_software/igenome.html. Accessed June 1, 2021.

[11] Li H., Handsaker B., Wysoker A., Fennell T., Ruan J., Homer N., Marth G., Abecasis G., Durbin R. (2009). S. genome project data processing, the sequence alignment/map format and SAMtools. Bioinformatics.

[12] Liao Y., Smyth G.K., Shi W. (2014). featureCounts: an efficient general purpose program for assigning sequence reads to genomic features. Bioinformatics.

[13] Robinson M.D., Oshlack A. (2010). A scaling normalization method for differential expression analysis of RNA-seq data. Genome Biol..

[14] Robinson M.D., McCarthy D.J., Smyth G.K. (2010). edgeR: a Bioconductor package for differential expression analysis of digital gene expression data. Bioinformatics.

[15] Shen S., Park J.W., Lu Z.X., Lin L., Henry M.D., Wu Y.N., Zhou Q., Xing Y. (2014). rMATS: robust and flexible detection of differential alternative splicing from replicate RNA-Seq data. Proc. Natl. Acad. Sci. USA.

[16] Robinson J.T., Thorvaldsdottir H., Winckler W., Guttman M., Lander E.S., Getz G., Mesirov J.P. (2011). Integrative genomics viewer. Nat. Biotechnol..

[17] Yeo G., Burge C.B. (2004). Maximum entropy modeling of short sequence motifs with applications to RNA splicing signals. J. Comput. Biol..

[18] Giudice G., Sanchez-Cabo F., Torroja C., Lara-Pezzi E. (2016). ATtRACT-a database of RNA-binding proteins and associated motifs. Database.

[19] Heinz S., Benner C., Spann N., Bertolino E., Lin Y.C., Laslo P., Cheng J.X., Murre C., Singh H., Glass C.K. (2010). Simple combinations of lineage-determining transcription factors prime cis-regulatory elements required for macrophage and B cell identities. Mol. Cell..

